# The Conventional Nature of Non-MHC-Restricted T Cells

**DOI:** 10.3389/fimmu.2018.01365

**Published:** 2018-06-14

**Authors:** Marco Lepore, Lucia Mori, Gennaro De Libero

**Affiliations:** Experimental Immunology, Department of Biomedicine, University of Basel and University Hospital of Basel, Basel, Switzerland

**Keywords:** CD1, MR1, lipid antigens, immunotherapy, vaccines

## Abstract

The definition “unconventional T cells” identifies T lymphocytes that recognize non-peptide antigens presented by monomorphic antigen-presenting molecules. Two cell populations recognize lipid antigens and small metabolites presented by CD1 and MR1 molecules, respectively. A third cell population expressing the TCR Vγ9Vδ2 is stimulated by small phosphorylated metabolites. In the recent past, we have learnt a lot about the selection, tissue distribution, gene transcription programs, mode of expansion after antigen recognition, and persistence of these cells. These studies depict their functions in immune homeostasis and diseases. Current investigations are revealing that unconventional T cells include distinct sub-populations, which display unexpected similarities to classical MHC-restricted T cells in terms of TCR repertoire diversity, antigen specificity variety, functional heterogeneity, and naïve-to-memory differentiation dynamic. This review discusses the latest findings with a particular emphasis on these T cells, which appear to be more conventional than previously appreciated, and with the perspective of using CD1 and MR1-restricted T cells in vaccination and immunotherapy.

## Introduction

T lymphocytes recognize complexes made by antigen-presenting molecules and small antigenic molecules. Small peptides generated after digestion of large proteins can form stable complexes with MHC molecules. Their generation may occur in different intracellular compartments, which are the stations where cellular protein degradation occurs. Short peptides usually associate with the MHC molecules that co-localize in the same cellular organelles.

T cells may also recognize non-peptide antigens, which are bound and presented by diverse non-polymorphic antigen-presenting molecules.

One group of T cells reacts to lipids, which form complexes with CD1 molecules. The four CD1 proteins with antigen presentation capability (CD1a, CD1b, CD1c, and CD1d) have different antigen-binding pockets and traffic alongside diverse intracellular routes. These features confer unique lipid-binding capacities to individual CD1 isoforms and allow them presenting a large variety of lipid molecules derived from microbes and plants or of self-origin.

Invariant natural killer T (iNKT) cells are the best-characterized CD1-restricted T cells. They are stimulated by the prototype lipid α-galactosylceramide (α-GalCer), by a variety of CD1d-presented lipid antigens made by several bacteria and also by peroxisome-derived self-lipids. Because they recognize conserved lipids shared among different microbes, they represent a large fraction of total T cells.

A second group of T cells recognizes small metabolites from the mevalonate pathway synthesized by APCs or of microbial origin. Human T cells expressing the TCR Vγ9/Vδ2 recognize APC accumulating endogenous isopentenyl-diphosphate or in the presence of microbial (E)-4-hydroxy-3-methyl-but-2-enyl diphosphate (1) and require the expression of butyrophilin 3A1 (2).

A population named mucosal-associated invariant T (MAIT) cells recognize transient metabolites generated during the microbial synthesis of riboflavin (vitamin B2) when associated with MHC-I-related molecule 1 (MR1) (3). MAIT cells react to a wide range of riboflavin-producing microbes and are abundant in human blood, liver, intestine, skin, and other organs (4). Table 1 summarizes some of their characteristics.

**Table 1:** MR1-restricted human T cells.

	TCRα	TCRVβ	Transcription factors	Phenotype	MR1 tetramer
Mucosal-associated invariant T (MAIT)	TRAV1-2/TRAJ33, TRAJ20, TRAJ12	TRBV6^a^, TRBV20-1^a^	PLZF, RORγt, T-bet	CD4 or CD8αα or CD8αβ, CD161^hi^, IL18Rα, CD26	5-OP-RU

Atypical MAIT	TRAV1-2	Polyclonal			5-OP-RU, 6-FP, Ac-6-FP

MR1T	Polyclonal	Polyclonal	PLZF, RORγt, T-bet	CD8 or CD4^-^CD8	Not done^b^

*^a^Preferential usage*.

*^b^Ags not identified*.

*5-OP-RU, 5-(2-oxopropylideneamino)-6-D-ribitylaminouracil; 6-FP, 6-formylpterin; Ac-6-FP, acetyl 6-formylpterin*.

An important feature of all these T cells is their capacity to promptly mediate diverse effector functions without the need for previous expansion after antigen recognition. Because of this ability, they are also defined as being innate-like T cells, as their immediate response mimics that of cells belonging to the innate immune compartment.

Innate-like T cells recognize almost ubiquitous and evolutionary conserved antigens, which are frequently encountered in the body, such as the aforementioned lipids and small metabolites. In addition, as is the case for iNKT and MAIT cells, they express a semi-invariant and oligoclonal TCR, in which a highly conserved TCR Vα chain is associated with a small number Vβ chains. In some cases, restricted amino acid use also occurs in the CDR3 regions of the α and β or γ and δ chains and it appears to be selected by antigen stimulation (4). Therefore, these cells evolved the capacity to recognize conserved microbial products, in some instances necessary for microbial survival and thus representing permanent microbial signatures.

The discovery of T cells reactive to non-protein antigens presented by non-polymorphic molecules, has prompted their definition as “unconventional,” with the aim of differentiating them from “conventional” peptide-specific T cells restricted to MHC molecules. This appellative, although helpful in distinguishing the two populations, may generate some confusion as the adjective “unconventional” is often used as synonym for innate-like T cells. Indeed, it is now clear that additional T cell populations exist in humans, which are unconventional in their capacity to recognize non-peptide antigens presented by non-polymorphic proteins, but nevertheless are conventional in their close similarity to adaptive MHC-restricted T cells. Non-peptidic-specific T cells, which are not iNKT, MAIT, or Vγ9Vδ2 cells, display a polyclonal TCR repertoire and recognize diverse antigens. In addition, they expand from a naïve pool upon antigen encounter, acquire diverse specialized functions after a few days of maturation, and give rise to memory responses, thus displaying a naïve-to-effector/memory differentiation dynamic. All of these characteristics are common to conventional peptide-specific T cells.

Heterogeneous non-innate-like T cells restricted to CD1 and recognizing diverse microbial and self-lipid antigens have been reported in many studies. TCR γδ cells reacting to lipids presented by group 1 CD1 molecules have been also identified. Finally, a new population of MR1-restricted T cells, which do not recognize riboflavin-related metabolites, has recently been isolated. These non-peptide-specific T cells, which are adaptive-like and MHC-unrestricted, remain poorly characterized and represent large populations of T cells not previously appreciated. This review will focus on the current knowledge of their features, role in immunity and diseases, and their potential applications in immunotherapy.

## Adaptive-Like T Cells Restricted to CD1

CD1 proteins display some unique structural characteristics, which make them specialized in presenting lipids rather than peptides to T cells (5, 6). The CD1 antigen-binding groove is bulky, with a volume ranging between 1,280 and 2,200 Å^3^ across individual CD1 isoforms (CD1b > CD1c > CD1d > CD1a) (7–10). The grove commonly contains two pockets, called A′ and F′ in analogy to the A and F pockets found in MHC-I. These pockets extend deeply within the molecules and allocate the acyl chains of lipid antigens (11). Indeed, they are almost entirely unexposed to the solvent surface and are lined with non-polar amino acids, which mediate hydrophobic interactions with the aliphatic tails of the antigens. The connection between this highly hydrophobic antigen-binding groove and the hydrophilic external environment is generally provided by a single surface portal (F′ portal) that allocates the polar head-group of the bound lipids, thereby making it available for TCR interaction, and allows lipid loading/exchange (4, 12–14).

Individual CD1 isoforms display substantial differences in the dimension and shape of their antigen-binding clefts (11). CD1a has the smallest groove, with the F′ pocket partially exposed to the external surface, probably to allow rapid lipid exchange (10, 15–17). CD1b shows an additional pocket (C′) and a tunnel (T′), which connects A′ and F′ clefts (7, 18–22). This conformation permits CD1b to bind lipids with very long acyl chains (23). In CD1c, two more portals are present (D′ and C′ portals), which allow additional access points for lipid molecules to the antigen-binding cavity (9, 24–26) and are likely to be responsible for the high flexibility and versatility of this CD1 isoform in presenting a wide range of lipid structures (27).

A large variety of structurally different self-, microbe-, and plant-derived lipids stimulate specific adaptive-like T cells when presented by CD1 proteins (4, 12, 14, 17, 24, 26, 28, 29). Generally, each of them preferentially binds one of the four CD1 isoforms equipped with antigen presentation capacity (CD1a, b, c, and d), although promiscuity is also observed (4, 12, 14, 17, 24, 26, 28–30). Several factors such as the unique variations in the architecture of individual CD1 isoforms, their diverse intracellular trafficking routes, their differential pH requirements for optimal loading and their co-localization with distinct lipid-remodeling enzymes and chaperon proteins, dictate the type and the size of lipid antigens they present to T cells (13, 31–34).

More than two decades ago, pioneer studies described human T cells reacting to CD1-expressing cell lines in the absence of foreign antigens (35, 36). At the same time, the novel T cell reactivity mediated by CD1 was attributed to the recognition of lipid rather than peptide antigens (37, 38). These early discoveries triggered incredibly intense and prolific research activity aimed at enumerating, characterizing, and classifying CD1-restricted lipid-specific T cells as well as understanding their immunological roles. The exclusive expression in mice and rats of the CD1d isoform, due to the lack of group 1 CD1 genes in these laboratory animals, focalized these studies on CD1d-restricted T cells, whose most abundant subset is innate-like iNKT cells (39). Invariant NKT cell antigen specificities, developmental programs, functional capacities, and impact in immunity have been extensively characterized in *in vitro* studies and in animal models and these findings currently feed clinical research aiming to assess their therapeutic potential [reviewed in Ref. (40–42)].

Additional T cells restricted to group 1 CD1 isoforms have been identified (28, 43–46), and they resemble conventional MHC-restricted T cells specific for peptide antigens in several aspects. For this reason, we define them here as adaptive-like.

CD1-restricted adaptive-like T cells can be divided into two groups, based on the source of their antigens. The first group includes T cells restricted to group 1 CD1 (CD1a, CD1b, and CD1c) and recognizing exogenous lipids derived from the cell wall of *M. tuberculosis* (43, 46). These T cells comprise diverse subsets that might be classified according to their TCR usage. The expression of a germline-encoded TRAV1-2/TRAJ9 TCR chain, conserved among individuals and preferentially paired with TRBV6-2, defines a population of mycolate-specific CD1b-restricted T cells called germline-encoded mycolyl-reactive (GEM), which is contained in the CD4^+^ T cell compartment (20, 47, 48). A second subset recognize glucose-monomycolates (GMM), also presented by CD1b, and has been named LDN5-TCR like, because the TCR Vα/Vβ pair found in the prototypic cell clone LDN5 (49) is frequent in this subset (48, 50). These cells display TCRs repertoire biased toward TRAV17 and TRBV4-1 chains, and diverse expression of the CD4 and CD8 co-receptors (48, 50). Additional *Mycobacterium*-reactive T cells include other CD1b-restricted T cells specific for mycolic acid (MA) (48) glycerol monomycolates (51), diacylated sulfoglycolipids (52, 53) and lipoglycans (54–56), CD1c-restricted T cells recognizing mycoketides (57, 58), and T cells stimulated by the lipopeptide dideoxymycobactin presented by CD1a (59). These T cells preferentially express the CD4 co-receptor and display a polyclonal TCR repertoire. Interestingly, they also include a small population of TCR γδ cells displaying the Vδ1 chain (46).

**Figure 1:**
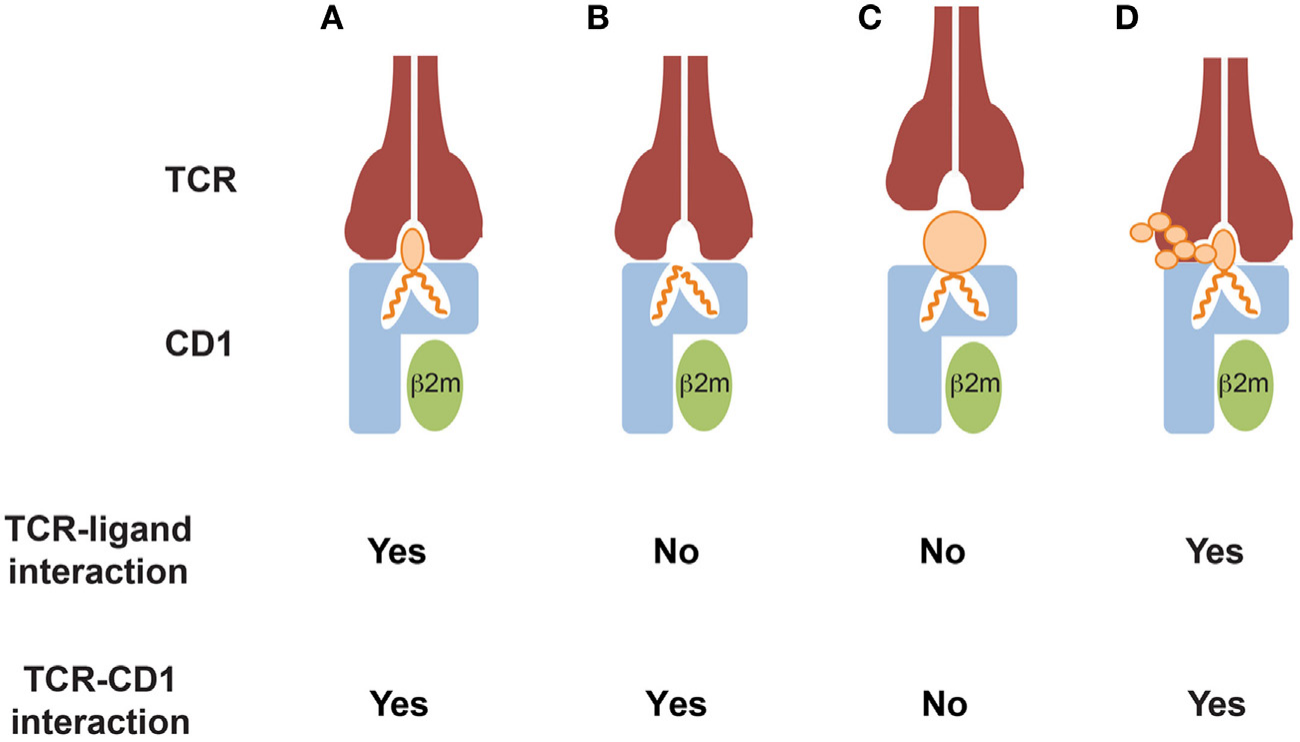
Modes of CD1-restricted TCR binding to CD1–lipid antigen complexes. **(A)** The TCR directly interacts with both the CD1 α1 and α2 domains and the bound lipid antigens. Key residues of the CDR3α and CDR3β loops directly contact the lipid antigens, allowing discrimination of small structural variations of their polar heads exposed to the solvent. **(B)** The TCR directly interacts with CD1 only and does not contact the lipid antigens. The antigens are often, but not always, headless lipids, which do not protrude out of the CD1 portals and probably induce small conformational changes favoring TCR binding. Lipid antigens that do not directly contact the TCR have been defined as “permissive.” **(C)** TCR binding is prevented by CD1 ligands that display large polar heads or contain solvent-exposed chemical groups that mediate repulsion with key residues of the TCR CDR3α and/or CDR3β loops. Ligands in this category have been defined as “non permissive.” **(D)** TCR binding occurs despite the presence of large and complex ligand polar heads, consisting of multiple sugar subunits. The TCR interacts with both CD1 and only a portion of the exposed lipid antigen head, which probably remains partially excluded from the binding surface area. This mode has not been supported by crystallographic studies, yet.

CD1-mediated T cell recognition of mycobacterial antigens occurs *via* direct and specific interaction of the TCR with the polar head of CD1-bound lipids (Figure 1A). Importantly, small variations in the structure or the stereochemistry of the lipid head-groups abrogate T cell recognition, thus supporting the fine antigen specificity of these T cells. For example, structural studies have demonstrated that a GEM TCR grasps the glucose ring of the GMM, acting like molecular tweezers (20). Interestingly, this TCR did not react to the same scaffold lipids displaying a mannose or a galactose instead of the glucose, suggesting that even small variations in the orientation of hydroxyl groups on the antigen head moiety, can strongly impact T cell reactivity (20). Similarly, CD1b-restricted T cells specific for the sulfoglycolipid Ac_2_SGL failed to recognize a version of this molecule devoid of the sulfate-group linked to sugar head-group, indicating an important role of this small moiety in mediating a direct interaction with the TCR (52). The size of the hydrophilic head is also important. A T cell clone specific for ganglioside GM1, which is made of four linear sugars and a branched sialic acid, did not recognize GM2 or GM3, which lack the terminal galactose of GM1 and the lateral sialic acid, respectively (Figure 1D) (60). Diverse mycoketide-specific T cells restricted to CD1c were also able to discriminate stereochemistry and structure alterations of their cognate antigens bound to CD1c (57, 58), thus further highlighting a remarkable fine specificity of these T cells.

A second group of adaptive-like CD1-restricted T cells recognizes target cells expressing CD1 isoforms in the absence of foreign antigens (28, 61). The autoreactivity of these T cells is due to the recognition of complexes formed by CD1 proteins and lipid molecules synthesized within APCs (28, 61). Diverse endogenous lipids including phospholipids, sphyngolipids, terpenes, and oils, stimulate group 1 CD1- and CD1d-restricted cells distinct from iNKT cells (17, 24, 28, 61).

The expression of CD4 and CD8 co-receptors and TCR repertoire are heterogeneous among these T cells (28, 61), although recent studies suggested that they might preferentially use recurrent TCR β chains. An example is provided by CD1c-autoreatcive T cells, which have recently been shown to be enriched in T cells expressing the TRBV4-1 or TRBV5-1 genes (26, 62). It is noteworthy that self-reactive CD1-restricted T cells include not only TCRαβ cells but also a small population of TCR γδ cells displaying the Vδ1 or Vδ3 chains (63–65).

**Figure 2:**
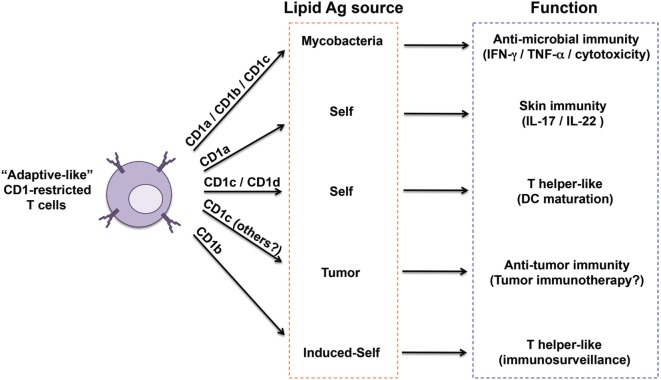
Heterogeneity of “adaptive like” CD1-restricted T cells. The sub-populations of CD1-restricted T cells reported so far as displaying close similarities to adaptive T cells are captured here in relation to their CD1 restriction, antigen sources, functions, and proposed immunological role. Invariant natural killer T cells have not been included because of their innate-like nature.

Two general mechanisms of antigen recognition have been described for these T cells. The first one relies on cognate interaction of TCR with the polar head of lipid antigens (Figures 1A,D). This has been observed for CD1a, CD1b, and CD1c-restricted T cell clones reacting to sulfatide (66) and CD1b-restricted clones responding to gangliosides (60). A second mechanism has been recently elucidated by crystallographic studies, which showed direct interaction between the TCR and the CD1 molecule but not with bound lipid antigens (Figures 1B,C). This mode of recognition was described for CD1a and CD1c-autoreactive T cells (16, 26), and implies that CD1 proteins bind “permissive” lipids. The presence in the CD1-binding pockets of such lipids might explain the observed frequent cross-reactivity toward diverse self-lipids, including headless molecules such as triglycerides, squalene, and cholesteryl esters. These important structural studies are uncovering key aspects of the interaction between TCRs and CD1-lipid complexes. However, a crystal structure is a snapshot of this interaction and is one of the many events required for T cell activation. Two aspects remain to be investigated: (i) whether recognition of permissive lipids requires additional signals provided by non-TCR molecules and (ii) whether different permissive lipids show a hierarchy of T cell-stimulatory potencies when APCs expressing physiological levels of CD1 molecules are tested. In addition, it will be very interesting to compare different autoreactive T cells and investigate whether unique endogenous lipids play major roles in the stimulation of these T cells in physiological and pathological settings. An example is provided by CD1c-restricted T cells recognizing methyl lysophosphatidic acids (mLPA), a newly defined lipid species accumulating in leukemia cells (67) (Figure 2). mLPA induced potent activation of specific CD1c-restricted T cells when exogenously added to CD1c^+^ B cells, which are not recognized in the absence of mLPA due to the scarce endogenous amounts of this lipid (67). Importantly, the same CD1c-restricted T cells strongly recognized and killed CD1c^+^ leukemia cells, which already have high mLPA quantities (67). These findings suggested that, at least in this case, mLPA, and not other permissive lipids, is the physiological antigen responsible for this reactivity (Figure 2).

A third type of CD1-restricted T cells shows characteristics of both groups described above. Indeed, they display dual reactivity toward self- and exogenous lipids derived from bacteria or plants (22, 30, 68). The basis of this cross-reactivity might rely on molecular mimicry or structural similarity between exogenous and endogenous stimulatory antigens.

The lack of group 1 CD1 genes in rodents limited the study of adaptive-like CD1-restricted T cell physiological functions and roles in diseases. *Ex vivo* data obtained with antigen-loaded CD1 tetramers and *in vivo* experiments in guinea pigs (that express several group 1 CD1 molecules) or in humanized mice, indicate that lipid-specific adaptive-like T cells participate in immunity against bacterial infections and that they might also be involved in autoimmunity and cancer (4) (Figure 2).

CD1-retricted T cells recognizing mycobacterial antigens were found expanded in *M. tuberculosis*-infected patients and in BCG-vaccinated individuals, supporting their adaptive-like properties and their role in protection (47, 51, 52, 59, 69, 70) (Figure 2). Furthermore, MA-specific T cells identified in the blood and lungs of tuberculosis (TB) patients displayed markers of effector and central memory cells, and persisted several months after successful treatment, indicating generation of a persistent memory T cell compartment (70). Lipoglycan-reactive T cells obtained from bronchoalveolar lavage of TB patients showed potent cytotoxic properties, and inhibited growth of intracellular mycobacteria (71). The recent detection of CD1b^+^ macrophages within lung granulomas of TB patients further suggests the importance of CD1-mediated immunity in this infection (72). In mice transgenic for the full human CD1 locus, both infection with mycobacteria or immunization with mycobacterial lipids elicited a slow primary CD1-restricted T cell response and very rapid secondary responses (73), similar to what was observed for peptide-specific T cells. Finally, studies in guinea pigs indicated that immunization with mycobacterial lipids or purified Ac_2_SGL conferred protections when challenged with *M. tuberculosis* (74–77).

The frequency of group 1 CD1-restricted T cells remains a poorly investigated issue. Two independent studies revealed that, in the blood of healthy donors, a surprisingly high frequency of T cells reacted to CD1-overexpressing targets in the absence of foreign antigens. A major fraction of these T cells were restricted to CD1a and CD1c molecules (78, 79). In one study, it was found that CD1-autoreactive T cells at birth were mainly contained in the naïve CD45RA^+^ compartment, while in adult blood their frequency among CD45RO^+^ effector/memory cells increased (78). These phenotypes are consistent with a progressive transition from naïve to effector/memory, typical of adaptive peptide-specific T cells. In addition, CD1a-restricted T cells expressed the skin-homing receptors CCR4 and CCR10 and could be isolated from skin biopsies (79). Their capacity to release IL-22 further suggested an immunological role in the skin (79, 80) (Figure 2). In addition, these autoreactive T cells could promote monocyte-derived dendritic maturation in a CD1c- or CD1d-dependent manner (81), thus attributing them a helper-like function (Figure 2). Self-reactive T cells might also act as sentinels for cell stress and inflammation. Indeed, APC may accumulate antigenic endogenous lipid antigens after microbial stimulation, and thus become very efficient in stimulating self-lipid-specific T cells (82) (Figure 2).

In individuals affected by Grave’s disease or Hashimoto thyroiditis, two autoimmune diseases of the thyroid, CD1a and CD1c self-reactive T cells infiltrated thyroid glands and were capable of lysing thyroid cells *in vitro* (83), possibly contributing to gland destruction. CD1c-autoreactive T cell clones isolated from systemic lupus erythematous (SLE) patients were able to provide pathogenic CD1c-dependent help to B cells *in vitro* (84). This study also showed that clones from healthy donors promoted IgM response in B cells, whereas cells isolated from SLE patients also elicited IgG production by the same B cells (84). These data suggested a role of CD1c self-reactive T cells in the genesis of the detrimental autoantibody responses that characterize this autoimmune disease. High frequency of circulating CD1-restricted T cells recognizing diverse self-glycosphingolipids were detected in multiple sclerosis patients (60, 66). Such clones preferentially recognized sulfatides made of long acyl chains, which are highly enriched in brain plaque lesions, thus showing a correlation between antigen specificity and lipid accumulation at sites of disease. Together these findings suggested that CD1-autoreactive T cells might participate in the pathologic process of myelin disruption.

CD1a-self-reactive T cells, which preferentially home to skin in healthy donors (79, 80), have been indicated as being capable of promoting inflammatory and autoimmune reactions of the skin (Figure 2). These cells accumulated in individuals with psoriasis and atopic dermatitis (85, 86). In both cases, the reactivity of these T cells depended on PLA2 secreted by mast cells in psoriatic lesions (85) or released by house dust mites in atopic dermatitis (86). PLA2 activity probably participates in generating CD1a-presented neoantigens, most likely lysophospholipids and free fatty acids, from the pool of skin phospholipids (85, 86). PLA2 is also a component of bee venom, and when injected sub-cutaneously induced local activation of CD1a-restricted polyclonal T cells (87, 88). In another study, urushiol, a molecule found in poison ivy and able to bind CD1a, induced CD1a-restricted T cells, which in a mouse model and in psoriasis patients amplified the local inflammation (17). Furthermore, contact sensitizers, including common cosmetic compounds, could unleash or potentiate the capacity of APCs to induce self-lipid-specific autoreactivity of skin-associated CD1a-restricted and CD1d-restricted T cells (89). All these data indicated CD1a, expressed at high levels on skin-resident Langerhans cells, as potential therapeutic target for skin inflammatory diseases.

## T Cells Restricted to MR1

A second population of T cells, which recognize non-peptidic antigens, is constituted by MR1-restricted T cells. The MR1 antigen-presenting molecule is also non-polymorphic and has structural similarities to MHC class I molecules as it is displayed on the cell surface as an heterodimer composed of a heavy chain non-covalently associated with β2 microglobulin (90). MR1 tissue distribution also resembles that of MHC class I molecules, as is almost ubiquitous (91), thus indicating that specific T cells might be activated by many different cell types.

MR1, like CD1 genes, is non-polymorphic. In addition and in contrast to CD1, the MR1 protein sequence is very conserved among species (91), which is not the case for CD1 genes. Both these features raise the question, what are the selection mechanisms that keep the MR1 structure conserved? Among the various possibilities, requirements for binding unique categories of antigens and/or a mandatory interaction with conserved molecules different from TCR might occur.

An intriguing MR1 feature is its antigen-binding pocket. This is formed by two interconnected cavities decorated by hydrophilic and hydrophobic aminoacids (92). The cavities are large enough to allocate molecules larger than the ones identified so far. Variability in ligand sizes might suggest that MR1 evolved the capacity of presenting antigens of different origins and chemical structure. This latter possibility is supported by the nature of the MR1-binding molecules that have been identified, so far. Among them are those formed by non-enzymatic condensation of 5-ribityl amino uracil, a precursor of riboflavin which is a typical bacterial molecule, with methylglyoxal or glyoxal carbonyls (3). Importantly, the resulting molecules activate MAIT cells in a very efficient manner. While recognition of microbial antigens by MAIT cells was anticipated by previous studies (93, 94), it was a surprise that the stimulatory antigens were neither peptides nor lipids. Structural studies also showed that formation of a covalent bond with MR1 is mandatory for stable binding and MAIT cell stimulation (3). Whether this bond has additional implications, such as a prolonged persistence of the MR1–antigen complex within APC and a reduced possibility of antigen exchange during MR1 recycling need further studies.

In another series of experiments, it was found that the MR1 antigen-binding pocket can also allocate small molecules, such as drugs, including salicylates and diclofenac, drug metabolites, and drug-like molecules (95). These compounds bound in different orientations, outlining the adaptability of the MR1-binding pocket. Intriguingly, all these molecules appeared to occupy only the A′ pocket of MR1, thus suggesting that bigger molecules might also occupy the F′ pocket.

**Figure 3:**
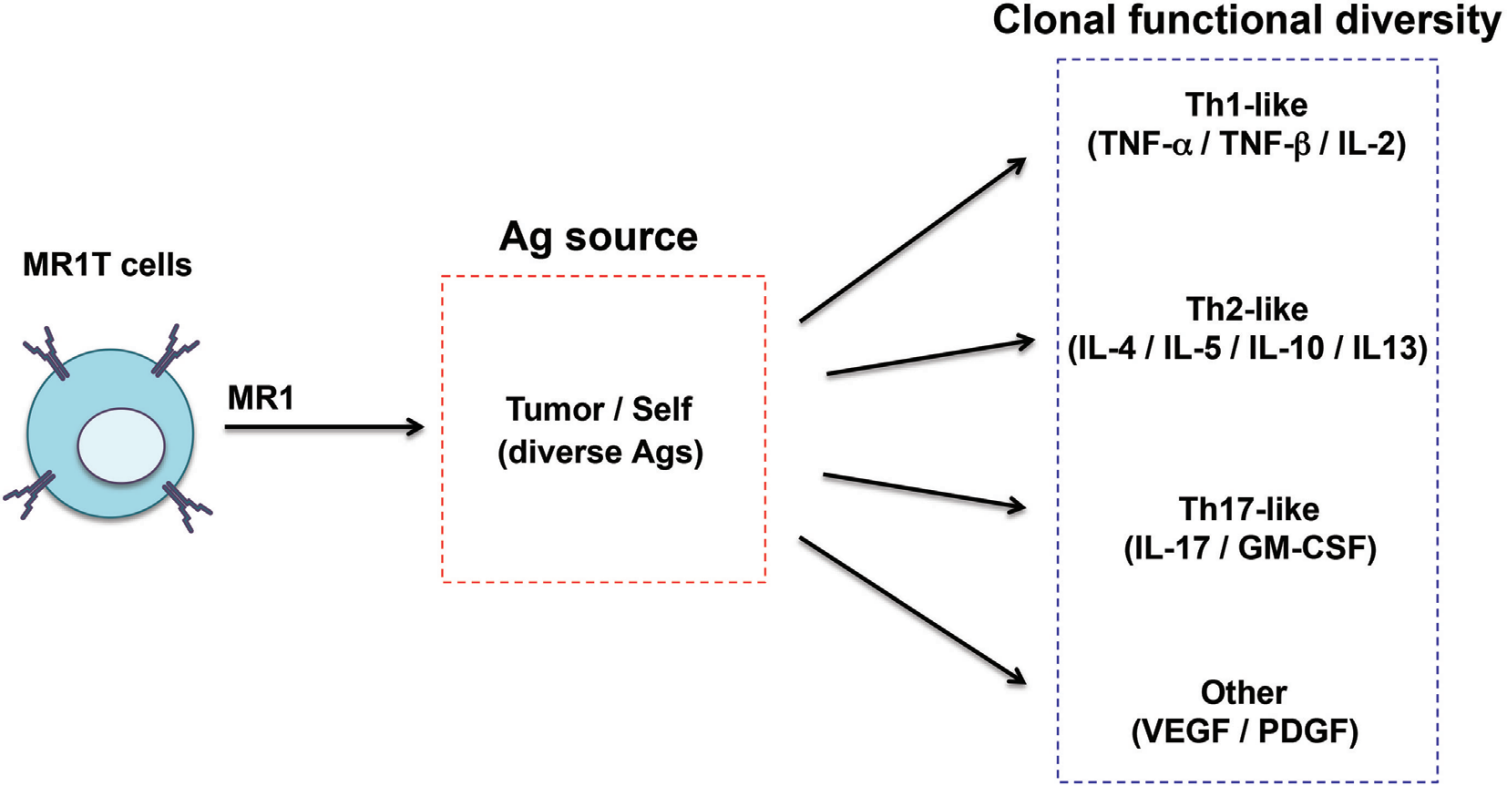
Functional diversity of MR1T cells. The heterogeneity of MR1T cells is illustrated in relation to the recognized antigen (yet unknown) and the functional phenotype of the clones isolated so far.

Recently, a second type of MR1-restricted T cells was identified. These cells, defined as MR1T cells, express polyclonal TCR chains, are CD8^+^ or CD4/CD8 double negative and can be isolated from circulating pool of healthy donors (96) (Table 1). MR1T cells showed preferential recognition of tumor cells and not of normal cells, even when the levels of expressed MR1 were physiologically very low. In addition, the presence of multiple antigens was suggested by differential recognition of tumor cells and by chromatographic separation of at least two antigens. Although the stimulatory molecules remain unknown, they are shared with mouse and hamster tumors. Upon antigen recognition, MR1T cells showed a variety of functions, including release of Th1 or Th2 cytokines, of vascular endothelial growth factor or platelet-derived growth factor AA, and expressed different transcriptional programs (96). These findings indicate that MR1T cells represent a novel population of functionally different T lymphocytes recognizing non-microbial antigens accumulating in tumor cells and showing characteristic of adaptive T cells (Figure 3).

As MR1T cells have been identified very recently, there is no literature concerning their role in diseases. The fact that these cells preferentially recognize tumor cells will promote new studies in patients harboring tumors expressing MR1 molecules.

In contrast, MAIT cells have been recognized for a decade and several studies have addressed their potential role in diseases. We briefly describe these findings below.

The identification of the bacterial antigens stimulating MAIT cells complemented a series of studies indicating a role of MAIT cells during bacterial infections. Both human and MAIT cells showed anti-mycobacterial effects (97, 98), and released a variety of cytokines upon recognition of bacteria-infected APC (97). MAIT cells accumulated in the lungs of mice infected with *Salmonella typhimurium*, upon stimulation with antigen and a toll-like receptor agonist and participated in local inflammation by secreting IL-17 and IFNγ (99). Studies in patients with severe bacterial infections showed an early decrease in MAIT cell count (100) and a significant positive correlation among non-streptococcal bacterial infections and MAIT depletion. Patients with persistent decreased MAIT cell numbers showed increased susceptibility to intensive care unit-acquired infections. A reduced number of circulating MAIT cells and their parallel increase in the lung were reported in patients with TB (93, 94). Reduced numbers of circulating MAIT cells were described in cystic fibrosis patients with *Pseudomonas aeruginosa* infection (101). In donors infected with *Plasmodium falciparum* sporozoites MAIT cells increased up to 6 months after infection clearance (102). A role of MAIT cell in protection during bacterial infection was also indicated by impaired protection during infection with *Francisella tularensis* in MAIT-depleted mice (103) and a positive effect in mice transgenic for a TRAV1-TRAJ33 TCR chain (93). However, in chronic infections MAIT cells might also participate in tissue lesions, as found in a mouse model of gastric infection with *Helicobacter pylori* (104). Their secretion of IL-17 and cytotoxic activity were considered important in establishing tissue lesions in this model.

Mucosal-associated invariant T cells might also contribute to immune response during viral infections. In patients with HIV infection the number of circulating MAIT cells varies to different extents (105–107). These effects might be ascribed to concurrent bacterial infections and migration to peripheral tissues.

A recent study also outlined a role of MAIT cells in graft versus host disease (GVHD) (108). MAIT cells accumulated in different GVHD target organs, contributed to intestinal mucosal integrity, released large amounts of IL-17, and limited the expansion of alloreactive T cells in the graft. As MAIT cells are much more abundant in humans than in rodents, it will be important to perform detailed studies in patients receiving bone marrow transplantation.

Mucosal-associated invariant T cells have also been studied in several autoimmune diseases. MR1-deficient mice develop severe experimental autoimmune encephalomyelitis (109) and also show loss of gut integrity and worsened diabetes (110). Several studies showed alterations in the number of circulating MAIT cells in patients with SLE (111), and in obese and type II diabetes patients (112). MAIT cells detected in psoriatic plaques (113) secreted large quantities of IL-17, thus implicating a possible role in local inflammation. In inflammatory bowel disease, circulating MAIT cells were diminished, while their number was increased in the inflamed versus healthy mucosal tissue (114). MAIT cells from colon released large amounts of IL-17 and IL-22. Combined, these findings indicate that MAIT cells may contribute to local inflammation in various diseases. They might become active upon TCR triggering or in response to local high levels if IL-12 or IL-18 (115, 116), thus behaving as amplifiers of inflammation also in the absence of antigen recognition.

## Exploitation of Non-Peptide-Specific T Cells in Therapy

Non-peptidic specific T cells are being used in different clinical settings. The recent explosion of T cell immunotherapy in cancer prompted exploitation of CD1-restricted T cells in tumor patients.

Invariant natural killer T cells were the first non-conventional T cell population utilized. Several clinical trials are being performed in cancer using α-GalCer, a strong agonist of iNKT cells [recently reviewed in Ref. (41, 42, 117)]. Other studies took advantage of the adjuvant capacity of iNKT cells and utilized soluble CD1d-α-GalCer complexes conjugated with tumor-specific antibodies (118), or α-GalCer conjugated with tumor-associated peptides (119, 120) or administration of dendritic cells pulsed with both α-GalCer and long peptides from the melanoma-associated NY-ESO-1 protein (121). These treatments induced significant amelioration both in animal models and in clinical settings.

An important therapeutic strategy is based on tumor-specific recognition by group 1 CD1-restricted T cells. As lipids and in particular glycosphingolipids, are altered in tumor cells (122), tumor-associated lipids are of immunotherapeutic interest. mLPA is an example as it is preferentially expressed in leukemic cells and is presented by CD1c (67). mLPA-specific T cells recognized and killed CD1c^+^ leukemia targets and limited leukemia cell spread in a mouse model (67). Immunotherapeutic strategies might be transfer of mLPA-specific TCR genes and vaccination with mLPA to expand specific T cells in leukemia patients. Important bonuses of these approaches, which are not applicable in case of peptide antigens, are (i) the non-polymorphic nature of CD1c, which allows application to the entire human population, and (ii) the lipid structure preservation under selective pressure. Whether additional tumor-associated lipids stimulate other CD1-restricted T cells remains a very important area of research.

MR1-restricted T cells might also be considered in tumor immunotherapy. The adjuvant properties of MAIT cells might be exploited similarly to iNKT cells, although no studies are available at present. MR1T cells might offer an additional possibility based on specific recognition of tumor-associated antigens (96). Transfer of MR1T TCR genes and vaccination with MR1T-stimulatory antigens might parallel the approaches using mLPA-specific T cells.

A large number of studies identified microbial lipids stimulating group 1 CD1-restricted T cells [reviewed in Ref. (4)] and suggested their use in novel vaccines. Two studies showed that mouse (123) or guinea pig (77) vaccination with group 1 CD1-binding lipids confers protection in *M. tuberculosis* infection models. These promising results, together with the finding that lipid-specific T cells expand in TB patients (52, 69) will prompt investigations to formulate novel lipid-based TB vaccines.

## Concluding Remarks

Non-peptide-specific T cells are abundant in blood, have a frequency similar to MHC-restricted T cells and can be considered important protagonists of adaptive immunity. Their functions, tissue distribution and capacity to generate memory populations are revealing unforeseen immunological roles. The identification of the antigenic repertoire stimulating both CD1- and MR1-restricted T cells will be instrumental to depict their participation in immune homeostasis and in diseases.

## Author Contributions

ML, LM and GDL wrote and revised the manuscript and approved its final version for publication.

## Conflict of Interest Statement

The authors declare that the research was conducted in the absence of any commercial or financial relationships that could be construed as a potential conflict of interest.
